# Novel predictions arise from contradictions

**DOI:** 10.1186/s13059-021-02371-6

**Published:** 2021-05-11

**Authors:** Itai Yanai, Martin Lercher

**Affiliations:** 1 Institute for Computational Medicine, NYU Langone Health, New York, NY 10016 USA; 2 Institute for Computer Science & Department of Biology, Heinrich Heine University, 40225 Düsseldorf, Germany

*“In formal logic, a contradiction is the signal of defeat, but in the evolution of real knowledge, it marks the first step in progress toward a victory.” Alfred North Whitehead*

From the outside, science seems to be the epitome of order, with its careful logical process, white lab coats, the methodical analyses of data, and, at its core, the formal testing of hypotheses. While that image may capture the “day science” aspect of science, it ignores the creative “night science” part, which generates the hypotheses in the first place. When we are in night science mode, we recognize facts that do not sit quite right against our clear and precise mental representation of the state of knowledge. While such contradictions arise from the data generated by day science, it is night science that revels in them, as these are the first, faint glimpses of new concepts. Depending on our state of mind, contradictions might appear as nuisances; embracing them helps us to counteract our natural human tendency for confirmation bias, a well-documented phenomenon in psychology. To explore the interaction of confirmation bias with a contradiction present in a dataset, we devised a simple experiment: individuals with different expectations examined the same data plot, which showed a superposition of two conflicting trends. We found that participants who expected a positive correlation between the two variables in the plot were more than twice as likely to report detecting one than those expecting a negative correlation. We posit that night science’s exploratory mode counteracts such cognitive biases, opening the door to new insights and predictions that can profoundly alter the course of a project. Thus, while science’s practitioners may be biased, the cyclical process of day science and night science allows us to spiral ever closer to the truth.

## Data is not transparent

Science prides itself on being above the “idols of the tribe and the cave” [1], unperturbed by assumptions and unproven theories. Data is objective, after all, and scientists commit to “letting the data do the talking.” But data, of course, cannot speak for itself. It must be interpreted against an extensive conceptual, theoretical, and methodological background—and this background is unlikely to be bias-free. Thus, to venture that a dataset makes a particular statement hides the degree to which potential biases may have influenced our conclusions.

**Fig. 1:**
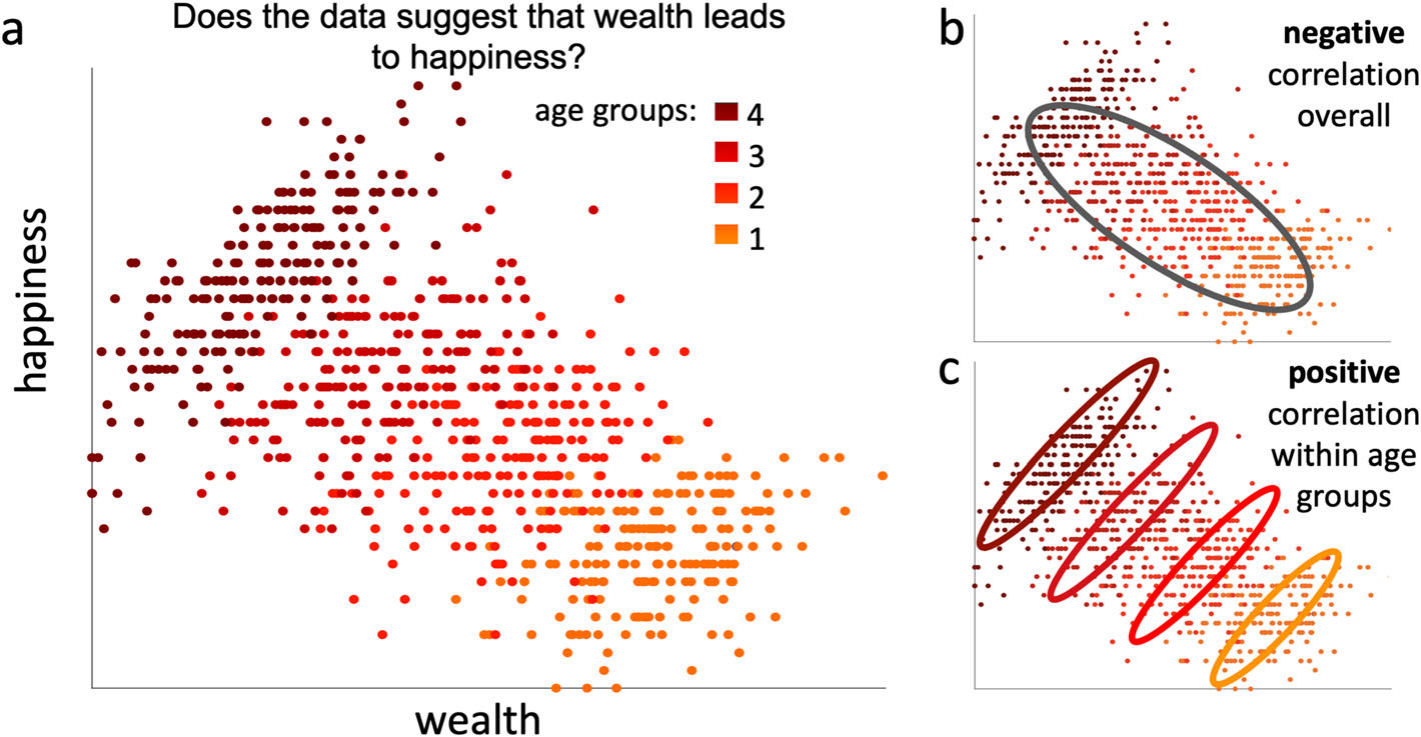
“Does the data suggest that wealth leads to happiness?” **a** Plot shown to participants on the alleged relationship between personal wealth and life satisfaction (“happiness”), where each point of the artificially created dataset represents one individual, colored by age group (1 oldest, 4 youngest). **b**, **c** Same as **a**, highlighting the overall negative correlation (**b**) and the positive correlations within age groups (**c**)

To study how our biases influence data interpretation, we performed an experiment with university students of computer science [2]. We showed them the plot in Fig. 1a, claiming that the data was collected to study the relationship between the personal wealth of individuals (*x*-axis) and their life satisfaction (“happiness,” *y*-axis). The data points indicating individuals were colored according to age groups. We then asked each participant: “Does the data suggest that wealth leads to happiness?”

The plot shows an overall negative correlation between wealth and happiness (Fig. 1b), while within each age group, a positive correlation is evident (Fig. 1c). This is an example of Simpson’s paradox [3, 4], where the correlation between two variables changes sign after controlling for another variable. The most parsimonious explanation of the pattern in the plot is that, all else being equal, more money makes you happier (thus the positive within-age group correlation). Across age groups, this effect is drowned out by a second, stronger effect from an underlying negative relationship between age and happiness. [We note that since this data was artificially created, no conclusions on real-life connections should be drawn.] In sum, while a first glimpse may indicate that wealthier individuals are less happy, the data indeed suggests that wealth leads to happiness. About a third of our participants acknowledged this by answering “yes” (49/171).

Regardless of what conclusion a particular participant made, we would like to assume that it arose from an analysis of the data plot, rather than resulting from preconceived notions. To test if this was indeed the case, we had inquired into the participants’ biases before showing them the data plot, asking the following question: “Imagine collecting data to study the relationship between the personal wealth of individuals and their life satisfaction (“happiness”). What overall general correlation do you expect?” Seventy percent of the students (119/171) expected a positive correlation, while the remaining students expected a negative one. Strikingly, the two groups saw the same data differently: those with an expectation of a positive correlation were more than twice as likely to conclude a positive correlation than those expecting a negative correlation (Table 1; 42/119 vs. 7/52, odds ratio 3.48, *P* = 0.0024 from one-sided Fisher’s exact test) [2].

**Table 1:**
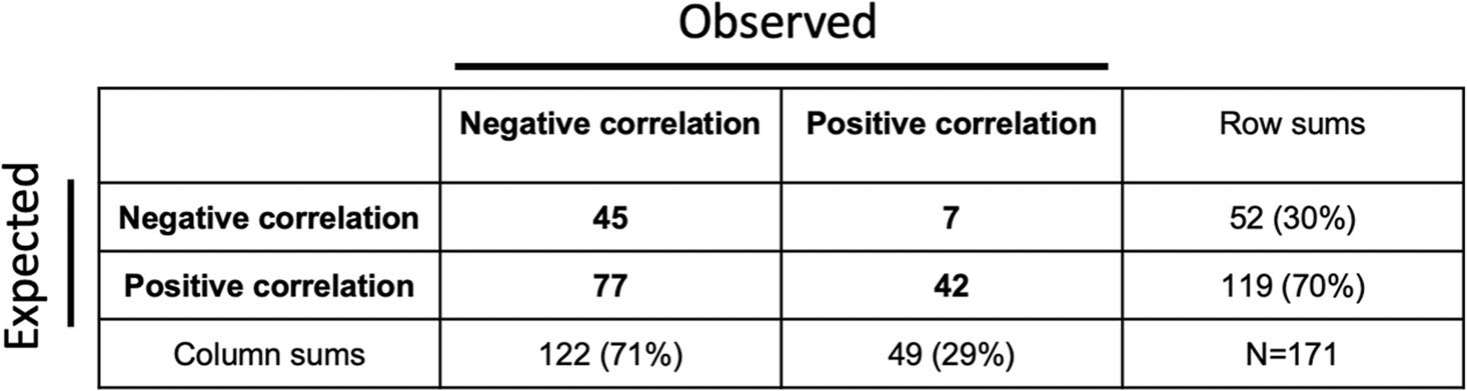
The contingency table for students in the two groups (expecting either a positive or a negative correlation between wealth and happiness) and what they observed [2]

Thus, the results suggest that when the participants looked at the plot, they came with a bias that framed their perception. This may reflect a general phenomenon in science. One famous episode stars Arthur Stanley Eddington, who in 1919 set out to test a prediction of Einstein’s theory of relativity. The data Eddington published could have been interpreted as either supporting Einstein’s or Newton’s theory of gravity (or, maybe most appropriately, as inconclusive). Yet in his paper, Eddington—who expected Einstein’s theory to prevail—framed the data as clearly supporting Einstein [5].

The extent to which the same data can lead to different conclusions has been the subject of recent studies that provide different scientific experts with a dataset together with a set of hypotheses to test. In one study, 70 independent teams were asked to analyze functional magnetic resonance images and to test 9 specific hypotheses [6]. Strikingly, no two teams chose identical work flows to analyze the dataset, and sets of teams reported contradictory, statistically significant effects based on the same dataset. In another study, 73 teams used the same data to test a single hypothesis; the “tremendous variation” in conclusions led the researchers to conclude a “vast universe of research design variability normally hidden from view” [7]. These and other studies [8–13] demonstrate that data is not transparent and that converting data into information through statistical analysis has a substantial subjective component. Our own experiment shows that even when studying the *same* plot, preconceived biases can result in different interpretations.

## Scientists are biased—especially you

Psychologists refer to “confirmation bias” as the propensity of viewing new evidence as supporting one's beliefs. Writing in the fifth century BC, the historian Thucydides put it this way: “It is a habit of mankind to entrust to careless hope what they long for, and to use sovereign reason to thrust aside what they do not fancy” [14]. In more recent times, experiments have shown that people demand a much higher standard of evidence for ideas they find disagreeable compared to those they hold themselves [15, 16]. As different individuals have different beliefs and experiences, each member of a community may end up perceiving reality differently, each through the prism of their own biased views. Confirmation bias may explain why harmful medieval medical practices were perpetuated over centuries, as only those patients that recovered (possibly despite rather than because of the treatment) were remembered [17]. The same phenomenon may underlie today’s widespread acceptance of “alternative” medicine [18–20].

While modern science appears to demand an objectivity that stands above such biases, confirmation bias is also well documented here. It shows itself, for example, in the peer review process of scientific papers. Studies whose findings are incompatible with a reviewer’s own assumptions are reviewed much more harshly than those that support the reviewer’s beliefs [21–23]. Confirmation bias leads scientists to dismiss or misinterpret publications that contradict their own preconceptions, up to the point where contradicting papers are cited as if they in fact supported a favored notion [24].

Beyond publications, does the execution of the scientific method itself also fall prey to confirmation bias? In theory, it should not, as emphasized by Karl Popper’s description of the scientific process [25]. For any given new idea, scientists should attempt with all of their experimental powers to falsify it, to prove it to be wrong. But, as philosopher Michael Strevens has argued, humans are not disciplined enough to strictly follow this method in their daily work [5]. Indeed, as much as we hold the philosophy of falsification in high regard, it simply is not very practical. First of all, hardly anyone actually excludes a beautiful and well evidenced hypothesis at the first sight of a falsifying result. If, for example, an experiment contradicts the conservation of energy, we do not simply throw out the first law of thermodynamics. Instead, we search for some flaw in the experiment or its interpretation [26]. But more than that, as we describe next, scientists—just like any other human beings—are hoping for and hence looking for evidence that confirms, not refutes, their favorite notions. If we make a prediction and the prediction seems to be borne out by the data on the surface, we tend to not dig deeper.

## Do not stop just because you like the result

Many scientists and the public at large are concerned about a “reproducibility crisis” in science: the observation that many published results cannot be replicated [27]. While it may be tempting to lay the fault at the door of a select group of misbehaving individuals, the wide scope of the observed non-reproducibility suggests that it may be a pernicious aspect of the scientific process itself [27].

As a point of reference, it is useful to recognize one scientific framework that escaped the reproducibility crisis: clinical trials. The acknowledgement and correction of potential biases is built into the rigid process of clinical trials, where study designs are pre-registered and detailed protocols have to be followed to the letter. Confounding variables are clearly identified a priori, the data is blinded to exclude potential biases of the practitioners, and significance is tested only when the pre-specified dataset has been collected. Clinical trials are carefully designed to be exclusively in the mode of hypothesis-testing; if executed with a large enough sample size, they ought to be immune not only to confirmation bias, but also to other sources of systematic irreproducibility.

In sharp contrast to the purely hypothesis-testing mode of a clinical trial, basic research projects typically do not fully know what to expect from the data before analyzing it—after all, “If we knew what we were doing, it wouldn’t be research, would it?” High-throughput datasets are especially likely to contain information unanticipated in their generation, observations one could not have predicted a priori; for this reason, we may often be better poised for a discovery if we conversed with a dataset without having formulated a concrete hypothesis [28]. The natural place for almost every dataset is right in the middle of the data-hypothesis conversation [29, 30], where it is used both in a hypothesis-testing way and in explorations that look for unexpected patterns [31], the seeds of future hypotheses.

Arguably, the central part of the scientific method is to challenge a hypothesis by contrasting its predictions with data. But when we do that, we are typically anxious for the falsification to fail: unless we are testing someone else’s competing hypothesis, we hope—and frequently expect—that our predictions will be borne out. If the results do not initially comply, we will think about problems with the experiment or with auxiliary assumptions (such as how we expect a genetic manipulation to perturb a cellular system). Sometimes, we will identify multiple such problems, and the results may converge to what we predicted them to be. There is, in principle, nothing wrong with this general approach: the scientific process is very much trial and error, and we cannot expect that an initial experiment and our first analysis were without fault. Obviously, we have to be careful not to selectively exclude contradictory data by making sure that similar errors have not equally affected other data points. There is a more subtle point here, though, a more discrete way in which confirmation bias may creep into our science. Our human instincts will lead to a sense of fulfillment once the expected pattern finally emerges. While this may mark the perfect time for a well-earned coffee break, it is not the time when we should stop analyzing the data. Instead, we should continue to think about possible biases and errors in our experiment, its analysis, and its interpretation. If we do not, we may be abandoning our efforts toward falsification too soon.

One extreme example, where confirmation bias is elevated to a guiding principle, is p-hacking [32]: one modifies the specifics of the analysis until the expected result emerges, subsequently reporting only the final configuration. It is important to realize, though, that in this case, it is the biased reporting that contributes to the reproducibility crisis, not the exploratory analysis itself [33]. Just the opposite: an exploration of how our results vary with changes in the specifics of the analysis, if communicated openly, provides important information on the robustness of our interpretation.

An elegant way to counter the drag toward self-fulfilling hypotheses is to test not one, but multiple alternative hypotheses, a core element of a method John Platt called “strong inference” [34]. Platt argued that the fastest scientific progress results from formulating a set of opposing hypotheses and then devising a test that can distinguish between them. While this is indeed a powerful approach, we often do not know initially what may be the best set of competing hypotheses. Forcing ourselves to look beyond one favored hypothesis in order to come up with such competing hypotheses is a serious—and non-fun—night science task, requiring hard and deliberate work.

## Embrace the contradiction

Looking back at the wealth-happiness experiment described above, the main aspect was also that the participants were faced with a contradiction. The data could be interpreted in two ways: a positive correlation if one looks at the data in one way (within age groups) or a negative correlation if one looks at it another (overall). Presumably, given enough staring at the data, each participant would have reached the same conclusion that it is the positive correlation that best summarizes the underlying relationship. As we argued above, the problem is that contradictions often go unexplored, perhaps because they are confusing or inconsistent with prior notions, or simply because acknowledging them suggests tedious additional work. And yet, a contradiction should be reason for joy: it hints at an apparent discrepancy between the state of knowledge and reality—we might have stumbled upon something new and interesting [35].

In research, we frequently find that to make sense of contradictory data, we must identify a false, hidden assumption. As we highlighted in an earlier piece [36], Einstein’s path toward the special theory of relativity began when he noticed a contradiction between Maxwell’s equations and the idea of traveling at the speed of light. It took him years of hard work, though, to identify the false, hidden assumption: that time was absolute and independent of our frame of reference.

As an example from our own work, in a recent project we compared developmental gene expression across ten species, each from a different phylum (flies, fish, worms, etc.) [37]. Having previously compared sets of species from the same phylum, we expected to again find a shared pattern of expression occurring toward the middle of the development of all embryos (the “hourglass model”). This was an exciting prospect: it would have confirmed a specific pattern of signaling and transcription factor expression common to all animals. However, when comparing the gene expression across phyla, what emerged was the exact opposite: the greatest correspondence between phyla occurred in the early and late transcriptomes, bridged by a less conserved transitional state (an “inverse hourglass”). Confused by this contradictory signal, we retreated into more night science to attempt a resolution. We finally realized that when combining the contradictory patterns within and across phyla, a molecular definition of animal phyla emerged: early and late development are broadly conserved, while the transitional state—conserved within, but highly variable between phyla—is phylum-specific, distinguishing one phylum from another. We had to learn to apply the two modes, the hourglass and the inverse hourglass, to different evolutionary timescales.

In the course of any project, there may be points where we stumble upon more or less blatant contradictions. The way in which we choose to deal with them will define the project’s fate. Embracing a contradiction requires us to be comfortable with uncertainty and will inevitably prolong the project. But it will provide space for unweaving the contradiction—arriving at the contradiction was not a signal of defeat, but rather the first sign of progress, as indicated by the Whitehead quote above.

In the absence of a contradiction, a common night science approach is to actually seek one out, playing devil’s advocate. Adopting a contrary viewpoint “for the sake of argument” can help to counter confirmation bias [38]. More than once, in a discussion with a student or collaborator, most of us have probably started a sentence with “Well, a reviewer might say….” If that does not help, a more severe approach would be to imagine that at some point in the future, a competing lab would write a paper that criticizes the current project. What would that “anti-paper” be about? By deliberately challenging our assumptions and expectations, we may avoid cheating ourselves out of discoveries.
